# Conflicts of interest and critiques of the use of systematic reviews in policymaking: an analysis of opinion articles

**DOI:** 10.1186/2046-4053-3-122

**Published:** 2014-11-18

**Authors:** Susan R Forsyth, Donna H Odierna, David Krauth, Lisa A Bero

**Affiliations:** 1 Department of Social and Behavioral Sciences, School of Nursing, University of California, San Francisco, CA, USA; 2 Department of Clinical Pharmacy, University of California, San Francisco, CA, USA; 3 Institute for Health Policy Studies, University of California, San Francisco, CA, USA; 4 Charles Perkins Centre, University of Sydney, Sydney, Australia

**Keywords:** Systematic reviews, Health policy, Conflicts of interest, Bias

## Abstract

**Background:**

Strong opinions for or against the use of systematic reviews to inform policymaking have been published in the medical literature. The purpose of this paper was to examine whether funding sources and author financial conflicts of interest were associated with whether an opinion article was supportive or critical of the use of systematic reviews for policymaking. We examined the nature of the arguments within each article, the types of disclosures present, and whether these articles are being cited in the academic literature.

**Methods:**

We searched for articles that expressed opinions about the use of systematic reviews for policymaking. We included articles that presented opinions about the use of systematic reviews for policymaking and categorized each article as supportive or critical of such use. We extracted all arguments regarding the use of systematic reviews from each article and inductively coded each as internal or external validity argument, categorized disclosed funding sources, conflicts of interest, and article types, and systematically searched for undisclosed financial ties. We counted the number of times each article has been cited in the “Web of Science.” We report descriptive statistics.

**Results:**

Articles that were critical of the use of systematic reviews (*n* = 25) for policymaking had disclosed or undisclosed industry ties 2.3 times more often than articles that were supportive of the use (*n* = 34). We found that editorials, comments, letters, and perspectives lacked published disclosures nearly twice as often (60% v. 33%) as other types of articles. We also found that editorials, comments, letters, and perspectives were less frequently cited in the academic literature than other article types (median number of citations = 5 v. 19).

**Conclusions:**

It is important to consider whether an article has industry ties when evaluating the strength of the argument for or against the use of systematic reviews for policymaking. We found that journal conflict of interest disclosures are often inadequate, particularly for editorials, comments, letters, and perspectives and that these articles are being cited as evidence in the academic literature. Our results further suggest the need for more consistent and complete disclosure for all article types.

## Background

Systematic reviews are often used to inform practice guidelines and public and private sector health policy decisions and are often used as an alternative to expert opinion or consensus conferences [1-4]. For example, systematic reviews have been used to guide policymaking around such issues as tobacco control and to set blood alcohol levels at which drivers are considered intoxicated [2]. States have used systematic reviews to evaluate whether policies for managing prison populations are working and cost effective [2]. Such use is not without controversy. While some authors argue that systematic reviews are the highest form of evidence and provide a solid basis for policymaking [1,2], others argue that these studies are methodologically flawed or limited in scope [5]. Editorials, letters, and other opinion pieces are important because they can inform debate about controversial topics. Furthermore, these articles are sometimes cited as if they are original research; a letter in the New England Journal of Medicine critical of a systematic review on secondhand smoke [5] has been cited 30 times as evidence of the flaws of systematic reviews, according to the Web of Science.

Systematic reviews synthesize the results of primary qualitative and quantitative research, using strategies to reduce bias [6,7]. Systematic reviews of qualitative research summarize and narratively synthesize results using a range of methods [8-10]. Meta-analyses are systematic reviews that summarize results quantitatively. This technique enables researchers to combine the results of several studies into a single effect estimate [11]. We use the term “systematic review” to refer to both systematic reviews and meta-analyses. Depending on the rigor of the protocol, the quality of the results of systematic reviews can be quite variable. High-quality reviews are characterized by a pre-defined protocol, clearly stated research questions, pre-defined eligibility criteria for studies, reproducible methodology, comprehensive search strategies to identify all studies and unpublished research that meet eligibility criteria, quality assessments including assessment of risk of bias, and systematic syntheses and presentation of findings [11]. Previous research has demonstrated that study results and conclusions may be influenced by funding sources and author conflicts of interest (COI) [12]. Primary studies or reviews funded by the pharmaceutical or tobacco industries are more likely to having findings or conclusions that favor the sponsor’s product than those funded by non-industry sources [12-14].

However, little research has been done on whether industry ties influence the conclusions of articles that contain opinions on the use of systematic reviews for health policy decisions. Therefore, we examined financial and other COI disclosed by authors of articles with opinions about the use of systematic reviews in policymaking. We investigated whether affiliations, funding sources, financial ties, and other COI of authors were associated with support or criticism of the use of systematic reviews. Further, we qualitatively summarized the types of arguments presented.

Despite the importance of knowing a study’s funding source and authors’ COI, it is often difficult to find complete information. Disclosure policies vary among journals and may be different for research articles and opinion pieces [15-17]. Therefore, we examined the extent of disclosure present in editorials, comments, letters, and perspectives as compared to other article types that expressed an opinion. In addition, authors’ COI statements and funding disclosures may not be entirely accurate [18,19]. To explore this, we searched for undisclosed industry ties, examining whether such ties were also associated with an article’s stance on the use of systematic reviews.

The primary objective of this study was to determine whether industry ties—disclosed or undisclosed—were associated with the conclusions of opinion articles on the use of systematic reviews for health policy decisions. Our secondary objectives were to determine the substance of the arguments that authors were using to support their positions and how disclosure policies vary by journal and by article type and to determine the extent that these articles are being cited in the scientific literature. This study was not intended to be a systematic review, as we did not undertake a comprehensive search with multiple databases, nor did we contact authors for unpublished papers. This is an analysis of a representative sample of available opinion articles.

## Methods

### Search strategy

Opinion pieces are often difficult to locate, are inconsistently indexed, and may have uninformative titles [20,21]. For our systematic investigation, we conducted a preliminary MEDLINE text search (“Systematic reviews for health policy decision making”) and consulted a reference librarian to further develop our search strategy. We then conducted four more searches of MEDLINE for all articles published between January 1, 1946, and July 31, 2010, with the following MeSH terms and topics: Meta-Analysis as Topic [Mesh] AND health policy [Mesh]; Review Literature as Topic [Mesh] AND health policy [MAJR]; Review literature as topic [Mesh] AND health policy [Mesh]; and “Meta-analysis” and “problem” as topics.

We included editorials, letters, commentaries, and other articles expressing opinions about the use of systematic reviews for policy. Research articles, including studies, reviews, and case examples, were included if the authors expressed an opinion on the use of systematic reviews in policymaking within the body of the article. For included papers, we examined the MEDLINE summary page for associated comments for possible inclusion into our sample. The purpose of this was to understand the debate surrounding a particular publication on the use of systematic reviews in policymaking. In addition, we also included relevant articles from our files.

### Selection criteria

Two researchers (SRF and DHO) screened search result titles to identify articles that appeared in peer-reviewed journals that criticized or supported the use of systematic reviews for making health policy decisions. Titles were excluded if they did not include at least one of the terms: systematic reviews, meta-analysis, evidence-based, Cochrane, medical effectiveness research, drug effectiveness review program, or policy and evidence. Titles were additionally excluded if they appeared to be entirely related to statistical methodology or handling of data or only related to the use of systematic reviews solely for clinical decision-making.

All other titles were included. Each author (SRF and DHO) independently screened each search and compared results. The abstracts and full text of all titles selected by either author were reviewed for inclusion. We also included relevant articles from the author’s files that did not appear in the original searches.

Two authors (SRF and DHO) independently screened the abstracts and/or full text of each included title. Discrepancies were resolved by consensus. We included articles that contained significant arguments for or against the use of systematic reviews in policymaking. We considered arguments to be significant if they did not focus solely on a single systematic review, but rather commented on whether systemic reviews did or did not have a role in policymaking. We excluded articles that made no mention or only a brief mention of the use of systematic reviews for policymaking, systematic reviews of a particular topic, tools or instructional articles, articles that were entirely clinically focused, and articles/abstracts that were not available in English or online.

### Coding of articles as supportive or critical of the use of systematic reviews to inform policy

We coded articles as supportive if they promoted well-conducted systematic reviews as a strong method for gathering, evaluating, and disseminating bodies of research. Articles that included suggestions for improvement but still advocated systematic reviews as an important knowledge generation tool for policymakers were also coded as supportive. We coded articles as critical if they argued that systematic reviews did not generate useful or accurate knowledge or argued that the method is currently too limited to be useful.

### Coding of individual arguments in each article

We initially coded each article inductively, abstracting each argument the authors made to support their positions. After analysis, we then thematically grouped similar arguments together for analysis and coded them as “internal reliability” arguments or “external validity” arguments using adapted definitions from previous studies [14,22]. Although each article was coded overall as supportive or critical, an individual article could contain both critical and supportive arguments or arguments about internal or external validity.

Two authors (DHO and SRF) coded in duplicate 14% (37/264) of the arguments. We agreed 92% (34/37) of the time. We resolved disagreements by consensus. One author (SRF) coded the remainder of the articles. We also abstracted illustrative quotes from the articles, qualitatively summarized the arguments, and conducted a content analysis on the abstracted arguments [23].

#### Internal reliability arguments

If the authors argued that a well-conducted systematic review was an internally reliable and valid method of collecting and analyzing data, the argument was coded as supportive of internal reliability. If the authors argued that systematic reviews were internally flawed because of issues of selection bias, misclassification, confounding, study heterogeneity, flaws in data pooling and data analysis, and publication bias, the article was coded as critical of internal reliability.

#### External validity arguments

If the authors argued that well-conducted systematic reviews were externally valid, useful for highlighting gaps in the literature, or a strong summary source of evidence useful in policy contexts, the argument was coded as a supportive external validity argument. If the authors argued that systematic reviews were not useful for highlighting gaps in the literature, a weak summary source of evidence and/or not useful in policy contexts, and/or not generalizable, the argument was coded as a critical external validity argument.

### Coding of disclosures

#### Affiliation of authors

We coded each included article by the author affiliations stated in the article. If authors claimed more than one affiliation, each type was coded.

#### Conflicts of interest of authors

We coded each article for COI by examining disclosures using categories adapted from the International Committee of Medical Journal Editors [24]. We considered study sponsorship/funding separately from COI. If there was an explicit COI statement contained within the article, it was considered to have a disclosed COI. We then divided COI statements into three categories: no disclosed COI, industry-related COI, and other, which included all non-industry-related COI such as a school or a government. We coded an article as having an industry-related COI if at least one author reported explicit financial ties to a for-profit industry. If an article did not have an explicit conflict of interest statement, we coded it as “no information provided.”

#### Funding sources of articles

We coded all articles by disclosed funding sources. If there was an explicit statement about funding, or that the article had no funding, the paper was considered to include disclosure of its funding source. We coded funding statements into five categories: self-funded or no funding, private non-profit funding, industry funding, government funding, and mixed funding. If an article did not have an explicit funding statement, we coded it as “no information provided.”

#### Industry ties

For analysis, we collapsed affiliations, COI, and funding sources into one dichotomous variable, “industry tie or no industry tie”. Our primary outcome was presence of industry ties by supportive or critical argument.

### Extent of disclosure

We abstracted the date of publication and extent of disclosure statement in each included article. Some journals have only recently required and/or printed COI statements and funding disclosure statements [17,25,26]. We identified current COI and funding disclosure policies for each of the journals in which our study articles appeared. Articles were coded into three categories: no disclosure of either COI or funding source, disclosure of COI or funding source, or disclosure of COI and funding source.

### Identification of undisclosed COI

For authors of articles with no disclosed industry ties, we searched MEDLINE for any articles they had published within the 3 years before publication of the included study article. We assessed the disclosures in these articles using criteria set forth by the International Committee of Medical Journal Editors [24]. We classified the disclosures of the authors as: (a) publication of a study sponsored by industry; (b) affiliation with industry at the time of publication, including, but not limited to, employment, board membership, etc.; (c) disclosure of a financial relationship to industry, including patents, stock, research funding, grants, gifts, consultancy, royalties, expert testimony, service on industry speaker’s bureau, payments for manuscript preparation or review, travel, lectures, etc., or other relevant financial activities/relationships [24]. If an industry tie was identified in an article published within the 3 prior years of the study article, we categorized the study article as having an undisclosed industry tie. We then discontinued our search for the remaining publications related to the study article, including publications by co-authors.

We also searched the Integrity in Science (ISS) database that contains over 4,000 scientists with corporate ties [27]. Up until 2009, when the database stopped collecting data, the researchers at ISS routinely scrutinized more than 200 science-based federal advisory committees for undisclosed conflicts of interest and monitored the media and scientific literature for failure to disclose. While the database is not comprehensive, it does provide an additional resource to examine corporate ties of scientists. If an author of a study article was listed as having an industry tie within the last 3 years in the ISS database, we categorized the study article as having an undisclosed industry tie.

### Citations of included articles in the academic literature

We searched the academic search engine “Web of Science” and recorded the number of citations for each article included in our study as of November 2013. We used this number as a proxy measure for the extent that the article was being used as evidence within the academic community.

### Analysis

After articles were selected for inclusion, each article was closely read by two authors (SRF and DHO) and a decision was reached about whether the article was supportive or critical of the use of systematic reviews in policymaking. Discrepancies were resolved by a third author (LAB). Using an author-generated data collection sheet, we coded full citation and article type, whether the article was supportive or critical of the use of systematic reviews to inform policy, individual argument types, affiliations of authors, stated conflicts of interest of all authors, funding source of articles, and extent of disclosure, as described below. Once all data from the article had been extracted, we then searched for undisclosed industry ties and individual journal disclosure policies. Finally, we identified the number of times each article had been cited in the academic literature.

All data were analyzed using descriptive statistics, as the use of inferential statistics was inappropriate due to the non-randomness of the sample.

## Results

**Figure 1:**
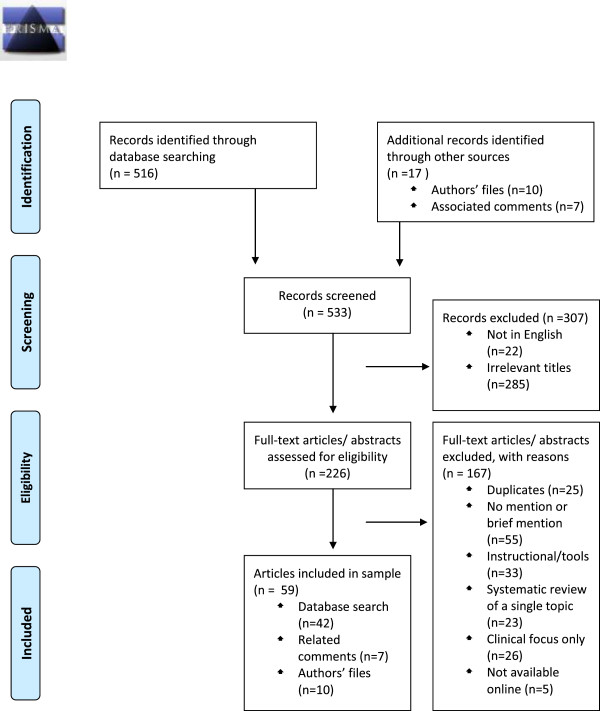
PRISMA 2009 flow diagram.

We screened a total of 533 article titles including ten from the author’s files and seven from comments associated with the articles (see Figure 1). Twenty-two articles were excluded because they were not in English (see Additional file 1). Of the 59 included articles (see Additional file 2), nearly 34% (20/59) of the included articles were editorials, comments, letters, and perspectives, while 66% (39/59) of the articles were research, reviews, and case studies. All included articles were published between 1991 and July 2010. We coded 58% (34/59) articles as supportive of using systematic reviews to inform policy and 42% (25/59) as critical.

### Disclosure of affiliation, conflict of interest, and funding source

Table 1 shows the extent of disclosure found in the study articles. Forty-two percent (25/59) of both supportive and critical articles contained no COI or funding source disclosures, whereas 21% (7/34) and 24% (6/25) of the articles, respectively, had both COI and funding disclosures. Disclosure status appeared to improve over time. The median publication date for articles without disclosures was 2003 for both supportive and critical articles, compared to 2008 for articles with COI and funding disclosures.

**Table 1:** Extent of disclosures made within the articles

**Extent of disclosure in study articles**	**Supportive papers**	**Critical papers**
	**(*n *** **= 34)**	**(*n *** **= 25)**
No disclosure of either conflicts of interest or funding source (%, *n*)	41 (14)	44 (11)
Median date	2003	2006
Date range	1995–2010	1991–2010
Disclosure of conflicts of interest or funding source (%, *n*)	38 (13)	32 (8)
Median date	2005	2005
Date range	2000–2009	1991–2010
Disclosure of conflicts of interest and funding source (%, *n*)	21 (7)	24 (6)
Median date	2008	2008
Date range	2005–2009	2003–2010

Table 2 shows the extent of disclosures found in the study articles by article type. Sixty percent (12/20) of the editorials, comments, letters, or perspectives did not contain either COI statements or funding disclosures, compared to 33% of the other article types considered.

**Table 2:** Extent of disclosures in editorials, comments, letters, and perspectives compared to other article types

	**Supportive**	**Critical**	**Total**
Extent of disclosure in editorials, comments, letters, and perspectives	(*n* = 8)	(*n* = 12)	(*n* = 20)
No disclosure of either conflicts of interest or funding source (%, *n*)	50 (4)	67 (8)	60 (12)
Disclosure of conflicts of interest or funding source (%, *n*)	37 (3)	17 (2)	25 (5)
Disclosure of conflicts of interest and funding source (%, *n*)	13 (1)	17 (2)	15 (3)
Extent of disclosures in other article types	(*n* =26)	(*n* =13)	(*n* =39)
No disclosure of either conflicts of interest or funding source (%, *n*)	38 (10)	23 (3)	33 (13)
Disclosure of conflicts of interest or funding source (%, *n*)	38 (10)	46 (6)	41 (16)
Disclosure of conflicts of interest and funding source (%, *n*)	23 (6)	31 (4)	26 (10)

Table 3 shows disclosed affiliations, COI, and funding sources of the study articles. We found similar rates of disclosed university affiliations among supportive and critical authors. However, more supportive authors were affiliated with government and considerably more critical authors were affiliated with the pharmaceutical, tobacco, or insurance industries, the three industries represented in our sample. The majority of papers did not have explicit COI statements. However, 26% (9/34) of the supportive authors explicitly stated that they did not have COI, compared to 8% (2/25) of the critical authors. Conversely, 32% (8/25) of the critical authors had an explicitly stated industry-related COI as compared to 3% (1/34) of the supportive authors. Funding statements were almost equally scarce among supportive and critical articles, and no article listed more than one funding source. Of those articles that disclosed funding sources, more articles critical of systematic reviews were funded by industry.

**Table 3:** Disclosed affiliations, conflicts of interest, and funding source by opinion on the use of systematic reviews for policymaking

	**Supportive articles**		**Critical articles**	
	**(*n *** **= 34)**		**(*n *** **= 25)**	
	**%**	***n***	**%**	***n***
Disclosed affiliations^a^				
University or university hospitals	85	29	76	19
Government	26	9	4	1
Non-profit	18	6	4	1
Non-university hospitals	9	3	8	2
Industry	0	0	20	5
Disclosed conflicts of interest—any author				
No conflicts of interests	26	9	8	2
Other (school, government)	6	2	0	0
Industry	3	1	32	8
Not disclosed	65	22	60	15
Disclosed funding source				
Self-funded/no funding	12	4	0	0
Private non-profit	12	4	0	0
Government	18	6	12	3
Industry	3	1	20	5
Mixed funding	0	0	0	0
Not disclosed	56	19	68	17

^a^Totals add to more than 100 as some papers had more than one affiliation.

### Industry ties—disclosed and undisclosed

Table 4 shows industry ties that were disclosed in the included article and industry ties that were found in the 3-year retrospective search for undisclosed ties. Critical articles were about six times more likely to have a disclosed industry tie than supportive articles; 6% (2/34) of supportive articles and 40% (10/25) of critical articles disclosed industry ties. While this gap narrowed when we included industry ties found in the retrospective 3-year search, critical articles had an industry tie more than twice as often as did supportive articles (35%, 12/34 v. 80%, 20/25).

**Table 4:** Industry ties (disclosed and not disclosed) by opinion on the use of systematic reviews for policymaking

**Industry ties**	**Supportive articles**		**Critical articles**	
	**(*n *** **= 34)**		**(*n *** **= 25)**	
	**%**	***n***	**%**	***n***
Industry ties disclosed in the article	6	2	40	10
Articles with industry ties not disclosed in the paper, but found on a 3-year retrospective search of previously disclosed industry ties in other articles	29	10	40	10
Total articles with industry ties (disclosed in the paper and those found on a 3-year retrospective search)	35	12	80	20

### Journal disclosure policies

At the time we conducted our study, most journals had current disclosure policies. The 15 articles critical of systematic reviews that did not disclose industry ties were published in 12 distinct journals. Among these journals, 9 of the 12 currently require authors to disclose their conflicts of interest and 6 of the 12 require authors to disclose their funding sources. The 32 supportive study articles that did not disclose industry ties were published in 20 distinct journals. Among these journals, 17 of the 20 currently require authors to disclose their conflict of interest and 13 of the 20 require authors to disclose their funding sources (data not shown in tabular form).

### Article citations

Table 5 shows the number of times that the included articles have been cited within the academic literature by article type and by whether the article was supportive or critical. Interestingly, 48% (12/25) of articles critical of systematic reviews were editorials, comments, letters, and perspectives, while only 24% (8/34) of supportive articles were editorials, comments, letters, and perspectives. Taken together, editorials, comments, letters, and perspectives were cited less often than other article types (median citations 5 v. 19); however, they still received a number of citations within the literature.

**Table 5:** Citations of editorials, comments, letters, and perspectives in the academic literature as compared to other article types (from the Web of Science by November 2013)

	**Supportive articles**	**Critical articles**	**Total articles**
Editorials, comments, letters, and perspectives	(*n* = 8)	(*n* = 12)	(*n* = 20)
Total number of citations	146	220	366
Average number of citations	18	18	18
Median number of citations	5.5	3	5
Range of citations	0–107	0–78	0–107
Other article types (reviews, research, and case studies)	(*n* = 26)	(*n* = 13)	(*n* = 39)
Total number of citations	1109	604	1713
Average number of citations	43	50	44
Median number of citations	19	21	19
Range of citations	0–490	5–220	0–490

### Argument analysis

#### Internal reliability arguments

We abstracted 151 individual arguments from the 34 supportive articles. Of these 151, 37% (56/151) were arguments in support of the internal reliability of well-conducted systematic reviews. Authors typically argued that the methods of well-conducted systematic reviews reduce bias. Lavis et al. contended that this lack of bias, as well the transparency and comprehensiveness of systematic review methodology, provides policymakers with more complete and valid information than they are likely to encounter in individual studies [28].

We abstracted 113 individual arguments from the 25 critical articles. Of these 113, 62% (70/113) were arguments that were critical of the internal reliability of systematic reviews. Typically, authors argued that the research studies used in systematic reviews are too dissimilar to be combined into a summary statement or statistic, included studies are cherry-picked; and that studies are excluded because of investigator bias, not because of inherent problems with the study. These authors argued that systematic reviews magnify publication bias or that they are often dependent on small numbers of underpowered and methodologically inadequate trials. Critical authors also contend that the methods used are not transparent, that research questions asked by systematic reviews are too narrowly focused to be of any use, or that meta-analyses may use analyses of information from subgroups collected after randomization, resulting in the possibility that the confounding variables may no longer be distributed at random. In a recent critical article that reviewed four published systematic reviews, the authors note that weaknesses encountered in many meta-analyses often stem not from the method itself, but from the poor design and reporting of the trials that make up the body of evidence available to answer the particular research question. They argued that this is exacerbated when the authors of meta-analyses fail to exclude poor-quality studies or to account for the variability in study quality by performing sensitivity analyses [29]. A commentary in the New England Journal of Medicine questioning the conclusions of several meta-analyses that found that secondhand smoke is harmful to health presented many common criticisms of these studies, including authors’ bias and lack of subject matter expertise, subject matter experts’ misunderstanding of meta-analytic methods, and such methodological flaws as failure to include relevant co-variables or account for heterogeneity or varying effect sizes in different populations [5].

#### External validity arguments

External validity arguments occurred almost twice as often in supportive arguments as did internal validity arguments (63% v. 37%). The authors of these articles generally argued that well-conducted systematic reviews were the highest level of evidence available [30,31] and effectively summarized large bodies of evidence, resulting in strong external validity [32]. Supportive authors also asserted that systematic reviews were useful in identifying gaps in the literature and highlighting research priorities.

Thirty-eight percent (43/113) articles critical of systematic reviews contained external validity arguments; however, they generally were extensions of arguments made that were critical of the internal reliability of systematic reviews, arguing that there can be no external validity because of the poor internal validity and reliability. For example, Eysenck [33] argued that the results of a meta-analysis on the toxicity of secondhand smoke by the National Research Council were scientifically meaningless and should not be used in policymaking because of detection bias resulting from unreliable cause of death information on death certificates, authors’ reliance on effect size, and exclusion of “evidence relevant to the paradigm of research.”

In five critical articles [34-38], and six supportive [30,39-43], authors expressed concerns about generalizability of systematic review results. The critical articles primarily argued that results were too general to be applied with confidence to individuals or typical patient populations and warned that the results might not capture complexities of disease and treatment or biological, environmental, and contextual variability. One [37] raised questions about the utility to physicians of summary statistics; another [36] described the process of pooling heterogeneous patient data as “the risks run in pooling data from different studies to determine care guidelines are enormous”. Two critical articles discussed social context [34,38]. Neumann et al. criticized the Drug Effectiveness Review Project, which produces systematic reviews of drug-drug comparisons that are used by some US states to develop Medicaid preferred drug lists for not adequately considering costs and a “full societal perspective” [34]. Most of the critical articles concluded that intractable flaws in systematic review methods greatly decreased their external validity and suggested that policymakers use caution and take other sources of information into account when making health policy decisions. While most of the critical articles did not raise issues of health equity, Ahmad et al. [38] expressed concerns about the use by public health policymakers in low-income countries of systematic reviews of studies that are conducted primarily in high-income countries. The authors were concerned that systematic reviews on HIV prevention and tobacco cessation lacked cultural and socioeconomic context, rendering the findings potentially less useful for developing policies and practice guidelines in low-income countries. The authors identified steps that systematic review authors could take to increase the strength of review-based recommendations for developing countries.

Six otherwise supportive articles also expressed concerns about generalizability. One [30] advised policymakers to consider contextual factors and study populations when applying systematic review results. Five articles [39-43] argued that while systematic reviews provided the best available evidence for development of health policies and practice guidelines, they did not adequately address issues of health equity or account for differences in socio-economic status, racial/ethnic diversity, and other social health determinants. All of these articles made recommendations to authors and policymakers for tools and approaches, many of them interdisciplinary, that they can use to enhance the utility of systematic reviews for the development of equitable health policy. One article advocating for evidence-based health promotion recommends that authors increase applicability of their reviews by taking into consideration the salience of the topic and outcome measures and the practical and cost implications of the interventions that are being considered [43]. Other authors stress the importance of providing credible evidence that is convincing within the health sector and also among non-health collaborators [40].

Articles without industry ties were more likely to accept systematic review methods as internally reliable and valid, focusing on the relevance of systematic reviews for policymaking, making arguments to support why and how policymakers should consider the results of systematic reviews in their policy decision-making. Articles that were critical of systematic reviews, but did not have industry ties, were more likely to make highly nuanced arguments expressing concern about the generalizability of systematic review results to diverse populations.

## Discussion

Our findings suggest that articles critical of the use of systematic reviews for policymaking are more likely to have industry ties than supportive articles. One possible explanation is that authors of well-conducted systematic reviews, such as those done by The Cochrane Collaboration, examine all of the data available about a topic, including unpublished data when possible, and select for inclusion studies that meet rigorous criteria [11]. Systematic reviews that are conducted according to strict pre-defined protocols leave industry with less ability to direct or criticize the review findings. By considering all of the available data, the summary effect estimate from a well-done systematic review should represent the current state of the science on the topic in question. Studies that are funded by industry are considered as part of the evidence, but usually not as the only evidence. This may leave industry with less control over the discourse, introducing variability and the possibility that their product, process, or service may not be the most efficacious. For example, unpublished data are more likely to show that a study drug is ineffective than data that are published [44-47]. Systematic reviews attempt to capture and include this information, thus leading to results that may ultimately conflict with the results of industry-sponsored randomized controlled trials.

Our findings also suggest that current journal disclosure requirements for articles are frequently inadequate, especially for editorials, comments, letters, and perspectives. These types of articles lacked any type of disclosure three times as often as the other article types. Editorials, comments, letters, and perspectives play an active role in the academic discourse, as evidenced by the number of times they have been cited in the literature. Together, the included articles have been cited 2,079 times in the literature with editorials, comments, letters, and perspectives cited 366 times. However, it should be noted that citations can either be favorable or unfavorable and that the total number of citations does not necessarily equate with the influence of the article.

Overall, 42% (25/59) of all the included articles, including some that were recently published, did not have any disclosures, leaving readers with no information on the author’s affiliations, funding sources, and COI. Others only had partial disclosures. Our detailed 3-year retrospective search found that 43% (20/47) of articles that had no disclosed industry ties had at least one author with an undisclosed industry tie from the previous 3 years. Having this information readily available is important when readers evaluate the strength of an argument and possible bias. By not requiring the information, or not printing it, journals may leave readers inadequately prepared to accurately judge an article’s accuracy or usefulness.

Our results are consistent with previous studies that showed a positive relationship between industry ties and favorable conclusions in research articles, even when the results of the study do not support the conclusions drawn [13,14,45,48]. Our research suggests that opinion pieces may be subject to similar biases as other article types and provides evidence that funding sources and COI for all article types should be made transparent.

Our research has several limitations. Because of the inherent difficulties of locating opinion articles, we acknowledge that it is likely that our list of included papers was not comprehensive. Rather, our aim was to perform a systematic search of the topic for a representative sample of articles. As our sample was not a random sample, we elected not to do inferential statistics with the results so that there would be no suggestion of false precision. Because of variable disclosure requirements of journals, we likely have under-estimated the number of industry ties in our sample. For example, one critical article, written during the debates over secondhand smoke [33], did not list any disclosures, nor did we find any industry ties during our 3-year retrospective search. This paper was catalogued in our study as not having any industry ties. However, a British newspaper [49] wrote that this particular researcher had received about 800,000 pounds from the tobacco industry. Finally, researchers who do not directly receive payments from industry may work at institutions in which industry funds infrastructure. We did not capture these relationships.

## Conclusions

Our findings are important because they demonstrate that industry ties may play a role in opinions expressed in the scientific literature. Moreover, opinion articles are often subsequently cited in the debate as evidence of the controversy over the use of systematic reviews in policymaking. Our results suggest the need for more consistent and complete disclosure for all article types, including articles that may not go through the traditional peer review process, such as editorials, commentaries, letters, and perspectives.

It is important to consider the industry ties of the authors when evaluating arguments regarding the use of systematic reviews by health policymakers and other decision makers. The conduct of systematic reviews is far from a perfect science, and there are substantial and nuanced criticisms of the generalizability of the results. While these arguments may be valid and should not be ignored, it is essential that we consider how knowledge generated by scientists can be critically summarized and subsequently translated for use in policymaking and population health decision-making. Well-conducted systematic reviews provide a rigorous and transparent method for knowledge dissemination and should be improved, not discarded.

## Competing interests

Susan Forsyth, Donna Odierna, and Lisa Bero are affiliated with The Cochrane Collaboration. The authors have no other competing interests to declare.

## Authors’ contributions

SRF assisted with the project development, collected and analyzed the data, and prepared drafts of the manuscript. DHO collaborated on the project development, developed the initial coding scheme, collected the data, wrote sections of the manuscript, and reviewed the drafts. DK collected the data and reviewed the drafts. LAB collaborated on the project development, contributed to the analysis of data, and reviewed the drafts. All authors read and approved the final manuscript.

## Supplementary Material

Additional file 1: **Study articles excluded for being in a language other than English.** Additional file 1 lists all studies from the original sample that were excluded because they were not in English. Click here for file

Additional file 2: **Included study articles.** Additional file 2 lists all studies included in the sample, including studies obtained from database searching, related commentary, and author’s files. Click here for file
