# Supplementary material for: Conflicts of interest and critiques of the use of systematic reviews in policymaking: an analysis of opinion articles
Source: Syst Rev. 2014 Nov 18;3:122. doi: 10.1186/2046-4053-3-122 (PMC4241194; doi:10.1186/2046-4053-3-122)
Supplement: Additional file 1: — Study articles excluded for being in a language other than English. Additional file 1 lists all studies from the original sample that were excluded because they were not in English. [file 2046-4053-3-122-S1.docx]

**Additional File 1**

1. Blettner M, Schlehofer B, Sauerbrei W: **[Limitations of meta-analysis from published data in epidemiological research].** *Sozial- und Praventivmedizin* 1997, **42:**95-104.

2. Bonfill X, Gabriel R, Cabello J: **[Evidence-based medicine].** *Revista espanola de cardiologia* 1997, **50:**819-825.

3. Carrillo-Gonzalez GM, Gomez-Ramirez OJ, Vargas-Rosero E: **[Meta-synthesis: a research methodology].** *Revista de salud publica (Bogota, Colombia)* 2007, **9:**609-617.

4. Cartabellotta A, Minella C, Bevilacqua L, Caltagirone P: **[Evidence-based medicine. 3. Systematic reviews: a tool for clinical practice, permanent education and health policy decisions. Italian Group on Evidence-Based Medicine-GIMBE].** *Recenti progressi in medicina* 1998, **89:**329-337.

5. Chabot JM: **[The WHO report and French government bill].** *La Revue du praticien* 2008, **58:**2039-2040.

6. Davila-Velazquez J, Martinez-Cairo S, Martinez-Garcia MC, Garduno-Espinosa J: **[Meta-analysis: an alternative method in clinical research].** *Boletin medico del Hospital Infantil de Mexico* 1991, **48:**576-582.

7. Degos F: **[Hepatitis B vaccine after the 2003 consensus conference].** *Gastroenterologie clinique et biologique* 2005, **29:**388-392.

8. Diener MK, Seiler CM, Antes G: **[Systematic reviews and meta-analyses in surgery].** *Der Chirurg; Zeitschrift fur alle Gebiete der operativen Medizen* 2007, **78:**938-944.

9. Egger M: **[Under the meta-scope: possibilities and limits of meta-analyses].** *Schweizerische medizinische Wochenschrift* 1998, **128:**1893-1901.

10. Gerhardus A, Muth C, Luhmann D: **[Adapting the "Curriculum of Evidence-based Medicine" to different target groups. Experiences with postgraduate studies in public health (Hanover) and medical education (Luebeck)].** *Zeitschrift fur arztliche Fortbildung und Qualitatssicherung* 2004, **98:**155-161.

11. Levy-Bruhl D: **[BCG Today].** *Presse Med* 2006, **35:**1733-1738.

12. Li YP, Tao TJ: **[Evidence-based medical approach to clinical medicine and health policy-making in the 21st century].** *Zhongguo yi xue ke xue yuan xue bao Acta Academiae Medicinae Sinicae* 2002, **24:**548-549.

13. Lu J, Shi L: **[Meta-analysis and its application in epidemiology].** *Zhonghua liu xing bing xue za zhi = Zhonghua liuxingbingxue zazhi* 1994, **15:**363-367.

14. Nony P, Boissel JP, Lievre M, Cucherat M, Haugh MC, Dayoub G: **[Introduction to meta-analytic methodology].** *La Revue de medecine interne / fondee par la Societe nationale francaise de medecine interne* 1995, **16:**536-546.

15. Sandholzer H, Hellenbrand W, Renteln-Kruse W, Van Weel C, Walker P: **[STEP--standardized assessment of elderly people in primary care].** *Deutsche medizinische Wochenschrift (1946)* 2004, **129 Suppl 4:**S183-226.

16. Silai R, Moussa M, Abdalli Mari M, Astafieva-Djaza M, Hafidhou M, Oumadi A, Randrianarivelojosia M, Said Ankili A, Said Ahmed B, Gayibor AH, et al: **[Surveillance of falciparum malaria susceptibility to antimalarial drugs and policy change in the Comoros].** *Bulletin de la Societe de pathologie exotique (1990)* 2007, **100:**6-9.

17. Subtil D, Truffert P, Vinatier D, Puech F, Querleu D, Crepin G: **[Value and limits of conventional or cumulative meta analysis in obstetrics and gynecology].** *Therapie* 1994, **49:**175-179.

18. Torregrossa MV: **[Biological and health effects on electric and magnetic fields at extremely low frequencies].** *Annali di igiene : medicina preventiva e di comunita* 2005, **17:**441-453.

19. van den Berg M, de Wit GA, Vijgen SM, Busch MC, Schuit AJ: **[Cost-effectiveness of prevention: opportunities for public health policy in the Netherlands].** *Nederlands tijdschrift voor geneeskunde* 2008, **152:**1329-1334.

20. Varela J: **[Consensus methodology in the health sector].** *Gaceta sanitaria / SESPAS* 1991, **5:**114-116.

21. Villers A, Grosclaude P, Haillot O, Abbou CC, Richard F, Boccon-Gibod L: **[Diagnosis of cancer of the prostate (I): Advancements in knowledge and practice since the consensus conference of 1989. The "Cancer of the Prostate" subcommittee of the Committee of Oncology of the French Association of Urology].** *Progres en urologie : journal de l'Association francaise d'urologie et de la Societe francaise d'urologie* 1997, **7:**508-515.

22. Vital Durand D: **[Problems raised by the conducting and interpretation of meta-analysis].** *Therapie* 1994, **49:**165-168.
