# Supplementary material for: Conflicts of interest and critiques of the use of systematic reviews in policymaking: an analysis of opinion articles
Source: Syst Rev. 2014 Nov 18;3:122. doi: 10.1186/2046-4053-3-122 (PMC4241194; doi:10.1186/2046-4053-3-122)
Supplement: Additional file 2: — Included study articles. Additional file 2 lists all studies included in the sample, including studies obtained from database searching, related commentary, and author’s files. [file 2046-4053-3-122-S2.docx]

**Additional file 2- Included study articles**

1. Ahmad N, Boutron I, Dechartres A, Durieux P, Ravaud P: **Applicability and generalisability of the results of systematic reviews to public health practice and policy: a systematic review.** *Trials* 2010, **11:**20.

2. Armstrong R, Doyle J, Waters E: **Cochrane Public Health Review Group update: incorporating research generated outside of the health sector.** *J Public Health (Oxf)* 2009, **31:**187-189.

3. Bailar JC, 3rd: **Passive smoking, coronary heart disease, and meta-analysis.** *N Engl J Med* 1999, **340:**958-959.

4. Bero LA, Jadad AR: **How consumers and policymakers can use systematic reviews for decision making.** *Ann Intern Med* 1997, **127:**37-42.

5. Bjordal JM, Bogen B, Lopes-Martins RA, Klovning A: **Can Cochrane Reviews in controversial areas be biased? A sensitivity analysis based on the protocol of a Systematic Cochrane Review on low-level laser therapy in osteoarthritis.** *Photomed Laser Surg* 2005, **23:**453-458.

6. Bolland MJ, Grey A, Reid IR: **Evidence from randomized controlled trials, meta-analyses, and subgroup analyses.** *JAMA* 2010, **303:**1254; author reply 1254-1255.

7. Bravata DM, McDonald KM, Shojania KG, Sundaram V, Owens DK: **Challenges in systematic reviews: synthesis of topics related to the delivery, organization, and financing of health care.** *Ann Intern Med* 2005, **142:**1056-1065.

8. Brownson RC, Fielding JE, Maylahn CM: **Evidence-based public health: a fundamental concept for public health practice.** *Annu Rev Public Health* 2009, **30:**175-201.

9. Chan KS, Morton SC, Shekelle PG: **Systematic reviews for evidence-based management: how to find them and what to do with them.** *Am J Manag Care* 2004, **10:**806-812.

10. Chaturvedi N, Bilous R, Hardy R, Remuzzi G, Ruggenenti P, Viberti GC: **Misleading meta-analysis: a need to look beyond the headlines.** *Diabet Med* 2007, **24:**587-591.

11. Chopra M, Munro S, Lavis JN, Vist G, Bennett S: **Effects of policy options for human resources for health: an analysis of systematic reviews.** *Lancet* 2008, **371:**668-674.

12. Coffman JM, Hong MK, Aubry WM, Luft HS, Yelin E: **Translating medical effectiveness research into policy: lessons from the California Health Benefits Review Program.** *Milbank Q* 2009, **87:**863-902.

13. Conn VS, Armer JM: **Meta-analysis and public policy: Opportunity for nursing impact.** *Nursing Outlook* 1996, **44:**267-271.

14. Cook DJ, Mulrow CD, Haynes RB: **Systematic reviews: synthesis of best evidence for clinical decisions.** *Ann Intern Med* 1997, **126:**376-380.

15. Cooper B: **Evidence-based mental health policy: a critical appraisal.** *British Journal of Psychiatry* 2003, **183:**105-113.

16. Cooper MJ, Zlotkin SH: **An evidence-based approach to the development of national dietary guidelines.** *J Am Diet Assoc* 2003, **103:**S28-33.

17. Coyne JC, Thombs BD, Hagedoorn M: **Ain't Necessarily So: Review and Critique of Recent Meta-Analyses of Behavioral Medicine Interventions in Health Psychology.** *Health Psychology* 2010, **29:**107-116.

18. Dobbins M, Thomas H, O'Brien MA, Duggan M: **Use of systematic reviews in the development of new provincial public health policies in Ontario.** *Int J Technol Assess Health Care* 2004, **20:**399-404.

19. Egger M, Smith GD: **Misleading meta-analysis.** *BMJ* 1995, **311:**753-754.

20. Enstrom JE, Kabat GC: **Environmental tobacco smoke and tobacco related mortality in a prospective study of Californians, 1960-98.** *BMJ* 2003, **326:**1057.

21. Enstrom JE, Kabat GC: **Environmental tobacco smoke and coronary heart disease mortality in the United States--a meta-analysis and critique.** *Inhal Toxicol* 2006, **18:**199-210.

22. Ernst E, Pittler MH: **Assessment of therapeutic safety in systematic reviews: literature review.** *BMJ* 2001, **323:**546.

23. Eysenck HJ: **Meta-analysis of best-evidence synthesis?** *J Eval Clin Pract* 1995, **1:**29-36.

24. Fielding JE, Briss PA: **Promoting evidence-based public health policy: can we have better evidence and more action?** *Health Aff (Millwood)* 2006, **25:**969-978.

25. Fleiss JL, Gross AJ: **Meta-analysis in epidemiology, with special reference to studies of the association between exposure to environmental tobacco smoke and lung cancer: a critique.** *J Clin Epidemiol* 1991, **44:**127-139.

26. Fox DM: **Evidence of evidence-based health policy: the politics of systematic reviews in coverage decisions.** *Health Aff (Millwood)* 2005, **24:**114-122.

27. Garner P, Gelband H, Graves P, Jones K, MacLehose H, Olliaro P, Cochrane Infect Dis G: **Systematic Reviews in Malaria: Global Policies Need Global Reviews.** *Infectious Disease Clinics of North America* 2009, **23:**387-+.

28. Goodman SN: **Have you ever meta-analysis you didn't like?** *Ann Intern Med* 1991, **114:**244-246.

29. Gruen RL, Morris PS, McDonald EL, Bailie RS: **Making systematic reviews more useful for policy-makers.** *Bull World Health Organ* 2005, **83:**480.

30. Heaton AH: **The DERP: is it an effective methodology?** *Health Aff (Millwood)* 2006, **25:**W276-278.

31. Hennekens CH: **Evidence from randomized controlled trials, meta-analyses, and subgroup analyses.** *JAMA* 2010, **303:**1253; author reply 1254-1255.

32. Hennekens CH, Demets D: **The need for large-scale randomized evidence without undue emphasis on small trials, meta-analyses, or subgroup analyses.** *JAMA* 2009, **302:**2361-2362.

33. Houde SC: **The systematic review of the literature: a tool for evidence-based policy.** *J Gerontol Nurs* 2009, **35:**9-12.

34. Laupacis A, Straus S: **Systematic reviews: Time to address clinical and policy relevance as well as methodological rigor.** *Annals of Internal Medicine* 2007, **147:**273-274.

35. Lavis JN, Posada FB, Haines A, Osei E: **Use of research to inform public policymaking.** *Lancet* 2004, **364:**1615-1621.

36. Law MR, Wald NJ: **Misleading meta-analysis. One incorrect meta-analysis does not invalidate them all.** *BMJ* 1995, **311:**1303.

37. Le Fanu J: **Misleading meta-analysis. Public policy is based on results of epidemiological meta-analyses that contradict common sense.** *BMJ* 1995, **310:**1603-1604.

38. LeVois ME, Layard MW: **Publication bias in the environmental tobacco smoke/coronary heart disease epidemiologic literature.** *Regul Toxicol Pharmacol* 1995, **21:**184-191.

39. LeVois ME, Layard MW: **Passive smoking and heart disease. Authors need to analyse the same data.** *BMJ* 1998, **317:**344; author reply 346.

40. Manchikanti L, Derby R, Wolfer L, Singh V, Datta S, Hirsch JA: **Evidence-based medicine, systematic reviews, and guidelines in interventional pain management: Part 7: systematic reviews and meta-analyses of diagnostic accuracy studies.** *Pain Physician* 2009, **12:**929-963.

41. Marini JJ: **Meta-analysis: convenient assumptions and inconvenient truth.** *Crit Care Med* 2008, **36:**328-329.

42. Maynard A: **Safety behind the veil of ignorance.** *Health Econ* 2003, **12:**1-2.

43. McElwee NE, Ho SY, McGuigan KA, Horn ML: **Evidence-based coverage decisions? Primum non nocere.** *Health Aff (Millwood)* 2006, **25:**W279-282.

44. Moreira T: **Entangled evidence: knowledge making in systematic reviews in healthcare.** *Sociol Health Illn* 2007, **29:**180-197.

45. Mowatt G, Grimshaw JM, Davis DA, Mazmanian PE: **Getting evidence into practice: the work of the Cochrane Effective Practice and Organization of care Group (EPOC).** *J Contin Educ Health Prof* 2001, **21:**55-60.

46. Mullen PD, Ramirez G: **The promise and pitfalls of systematic reviews.** *Annu Rev Public Health* 2006, **27:**81-102.

47. Neumann PJ, Drummond MF, Jonsson B, Luce BR, Schwartz JS, Siebert U, Sullivan SD: **Are Key Principles for improved health technology assessment supported and used by health technology assessment organizations?** *Int J Technol Assess Health Care* 2010, **26:**71-78.

48. Odierna DH, Bero LA: **Systematic reviews reveal unrepresentative evidence for the development of drug formularies for poor and nonwhite populations.** *J Clin Epidemiol* 2009, **62:**1268-1278.

49. Petticrew M, Tugwell P, Welch V, Ueffing E, Kristjansson E, Armstrong R, Doyle J, Waters E: **Better evidence about wicked issues in tackling health inequities.** *J Public Health (Oxf)* 2009, **31:**453-456.

50. Pick W: **Lack of evidence hampers human-resources policy making.** *Lancet* 2008, **371:**629-630.

51. Russell R, Chung M, Balk EM, Atkinson S, Giovannucci EL, Ip S, Lichtenstein AH, Mayne ST, Raman G, Ross AC, et al: **Opportunities and challenges in conducting systematic reviews to support the development of nutrient reference values: vitamin A as an example.** *Am J Clin Nutr* 2009, **89:**728-733.

52. Rychetnik L, Wise M: **Advocating evidence-based health promotion: reflections and a way forward.** *Health Promot Int* 2004, **19:**247-257.

53. Sackett DL: **A science for the art of consensus.** *J Natl Cancer Inst* 1997, **89:**1003-1005.

54. Shadish WR: **Introduction: The perils of science in the world of policy and practice.** *Health Psychol* 2010, **29:**105-106.

55. Shojania KG, Sampson M, Ansari MT, Ji J, Doucette S, Moher D: **How quickly do systematic reviews go out of date? A survival analysis.** *Ann Intern Med* 2007, **147:**224-233.

56. Sylvester R, Collette L, Duchateau L: **The role of meta-analyses in assessing cancer treatments.** *Eur J Cancer* 2000, **36:**1351-1358.

57. Tobin MJ, Jubran A: **Meta-analysis under the spotlight: focused on a meta-analysis of ventilator weaning.** *Crit Care Med* 2008, **36:**1-7.

58. Tugwell P, Robinson V, Grimshaw J, Santesso N: **Systematic reviews and knowledge translation.** *Bull World Health Organ* 2006, **84:**643-651.

59. Volmink J, Siegfried N, Robertson K, Gulmezoglu AM: **Research synthesis and dissemination as a bridge to knowledge management: the Cochrane Collaboration.** *Bull World Health Organ* 2004, **82:**778-783.
